# The 2020 Ming K. Jeang awards for excellence in *Cell & Bioscience*

**DOI:** 10.1186/s13578-021-00727-w

**Published:** 2021-12-14

**Authors:** Yun-Fai Chris Lau

**Affiliations:** University of California, San Francisco, USA

## Abstract

Three articles published by the research groups led by Yun-Bo Shi of the National Institute of Child Health and Human Development, National Institutes of Health, USA; Aria Baniahmad of the Institute of Human Genetics, Jena University Hospital, Germany; and Kuanyu Li of the Nanjing University Medical School, China, have been selected as the recipients of the 2020 Ming K. Jeang Award for Excellence in Cell and Bioscience.

We are delighted to announce the 2020 recipients of the Ming K. Jeang Award for Excellence in *Cell & Bioscience*. Three research groups, who each published an outstanding research article in *Cell & Bioscience* in 2020 [1–3], have been selected to receive this prestigious award by a panel of Editors, chaired by Dr. Ying E. Zhang. The Ming K. Jeang Awards for Excellence in *Cell & Bioscience* was established in 2011 with a generous endowment from the Ming K. Jeang Foundation to honor outstanding research articles published in *Cell & Bioscience*, the society journal of the Society for Chinese Bioscientists in America (SCBA), a non-profit scientific society, based in North America, https://scbasociety.org/. The selected articles are listed as below.

1. Thyroid hormone receptor beta is critical for intestinal remodeling during *Xenopus tropicalis* metamorphosis. Yuki Shibata, Yuta Tanizaki and Yun‑Bo Shi. *Cell & Bioscience* (2020) 10:46.

2. Senolytic compounds control a distinct fate of androgen receptor agonist and antagonist‑induced cellular senescent LNCaP prostate cancer cells. Thanakorn Pungsrinont, Malika Franziska Sutter, Maren C. C. M. Ertingshausen, Gopinath Lakshmana, Miriam Kokal, Amir Saeed Khan and Aria Baniahmad. *Cell & Bioscience* (2020) 10:59.

3. Iron accumulation in macrophages promotes the formation of foam cells and development of atherosclerosis. Jing Cai, Meng Zhang, Yutong Liu, Huihui Li, Longcheng Shang, Tianze Xu, Zhipeng Chen, Fudi Wang, Tong Qiao and Kuanyu Li. *Cell & Bioscience* (2020) 10:137.

Congratulations to these three groups of investigators for publishing their outstanding research results in *Cell & Bioscience* and winning the 2020 Ming K. Jeang Award.

We are looking forwards to receiving contributions of outstanding research articles and reviews from the scientific community in 2020 and beyond.
